# Calorie restriction during gestation impacts maternal and offspring fecal microbiome in mice

**DOI:** 10.3389/fendo.2024.1423464

**Published:** 2024-10-04

**Authors:** Stephanie P. Gilley, Meghan L. Ruebel, Sree V. Chintapalli, Clyde J. Wright, Paul J. Rozance, Kartik Shankar

**Affiliations:** ^1^ Department of Pediatrics, Section of Nutrition, University of Colorado School of Medicine, Aurora, CO, United States; ^2^ Microbiome and Metabolism Research Unit (MMRU), United States Department of Agriculture - Agricultural Research Service (USDA-ARS), Southeast Area, Little Rock, AR, United States; ^3^ Arkansas Children’s Nutrition Center, University of Arkansas for Medical Sciences, Little Rock, AR, United States; ^4^ Department of Pediatrics, University of Arkansas for Medical Sciences, Little Rock, AR, United States; ^5^ Department of Pediatrics, Section of Neonatology, University of Colorado School of Medicine, Aurora, CO, United States

**Keywords:** microbiome, fetal growth restriction, calorie restriction, pregnancy, growth

## Abstract

**Background:**

Maternal undernutrition is the most common cause of fetal growth restriction (FGR) worldwide. FGR increases morbidity and mortality during infancy, as well as contributes to adult-onset diseases including obesity and type 2 diabetes. The role of the maternal or offspring microbiome in growth outcomes following FGR is not well understood.

**Methods:**

FGR was induced by 30% maternal calorie restriction (CR) during the second half of gestation in C57BL/6 mice. Pup weights were obtained on day of life 0, 1, and 7 and ages 3, 4 and 16 weeks. Fecal pellets were collected from pregnant dams at gestational day 18.5 and from offspring at ages 3 and 4 weeks of age. Bacterial genomic DNA was used for amplification of the V4 variable region of the 16S rRNA gene. Multivariable associations between maternal CR and taxonomic abundance were assessed using the MaAsLin2 package. Associations between microbial taxa and offspring outcomes were performed using distance-based redundancy analysis and Pearson correlations.

**Results:**

FGR pups weighed about 20% less than controls. Beta but not alpha diversity differed between control and CR dam microbiomes. CR dams had lower relative abundance of *Turicibacter*, *Flexispira*, and *Rikenella*, and increased relative abundance of *Parabacteroides* and *Prevotella*. Control and FGR offspring microbiota differed by beta diversity at ages 3 and 4 weeks. At 3 weeks, FGR offspring had decreased relative abundance of *Akkermansia* and *Sutterella* and increased relative abundance of *Anaerostipes* and *Paraprevotella*. At 4 weeks, FGR animals had decreased relative abundance of *Allobaculum*, *Sutterella*, *Bifidobacterium*, and *Lactobacillus*, among others, and increased relative abundance of *Turcibacter*, *Dorea*, and *Roseburia*. Maternal *Helicobacter* abundance was positively associated with offspring weight. *Akkermansia* abundance at age 3 and 4 weeks was negatively associated with adult weight.

**Conclusions:**

We demonstrate gut microbial dysbiosis in pregnant dams and offspring at two timepoints following maternal calorie restriction. Additional research is needed to test for functional roles of the microbiome in offspring growth outcomes.

## 1 Introduction

Fetal growth restriction (FGR), also called intrauterine growth restriction, is the failure of a fetus to meet its growth potential (1, 2). FGR impacts up to 20% of pregnancies in low- and middle-income countries and contributes to both long- and short-term morbidity and mortality (3–6). There are many etiologies of FGR including maternal high blood pressure, preeclampsia, cigarette use, and genetic conditions (5, 7). However, maternal malnutrition is the most common preventable cause worldwide (8). FGR increases morbidity and mortality during infancy, as well as contributes to adult-onset diseases including obesity, chronic kidney disease, metabolic syndrome, and type 2 diabetes (3, 6). Multiple mechanisms contribute to these adverse outcomes such as epigenetic changes leading to altered gene expression (6, 9), decreased lean muscle mass at birth (10, 11), impaired nephron development (12), and diminished pancreatic beta cell mass (13).

Emerging evidence from our group and others suggests a role for the gut microbiome in the pathogenesis of FGR-related adverse outcomes. Multiple studies in mice, rats, pigs, and humans have shown gut microbiome dysbiosis in FGR offspring compared to normal weight controls (14–17). The microbiome plays many roles in overall health including education and modulation of the immune system (18–20), especially important for infants with FGR who are more susceptible to neonatal infections (3, 7). The microbiome may also impact feeding tolerance and development of necrotizing enterocolitis, both conditions with higher prevalence in FGR infants (7, 21–23). The gut microbiome, even in the earliest stages of life, can also predict the development of rapid weight gain, excess adiposity, and childhood obesity (24–27). Microbiome-directed interventions have been shown in multiple studies to influence growth in infancy and childhood. For example, neonatal antibiotic exposure is associated with reduced stature and increased risk of obesity (28, 29). Recent studies demonstrate a direct effect of the infant gut microbiome on intestinal barrier function and neuro-endocrine signaling (25). Microbial dysbiosis may therefore have long lasting effects on health after fetal growth restriction.

There is also increasing evidence that multiple maternal microbiomes impact pregnancy outcomes (30, 31). Fetal growth, establishment of the offspring microbiome, and development of the offspring immune system all depend on the maternal microbiome (14, 24, 32–36). Maternal microbial products such as lipopolysaccharide and metabolites including short-chain fatty acids reach the fetus and influence pregnancy outcomes including fetal growth and ultimately birth weight (33, 37, 38). The maternal gut microbiome changes during pregnancy and is further altered in the context of complications known to impact fetal growth and development, including gestational diabetes, preeclampsia, obesity, and fetal growth restriction (15, 24, 34, 39–43). However, the role of the gut microbiome in the setting of maternal calorie restriction in the pathogenesis of fetal growth restriction or early offspring growth is not fully understood.

In the present study we sought to examine the role of maternal caloric restriction on the maternal gut microbiome at the end of pregnancy (gestational day 18.5) and early offspring gut microbiome (postnatal days 21 and 28). In addition, we investigated potential associations of maternal taxa and maternal-fetal outcomes, as well as offspring taxa with early life weight and weight gain.

## 2 Methods

### 2.1 Ethical approval

All animal experiments and procedures were approved by the University of Colorado Institutional Animal Care and Use Committee (AUP#00274) and conducted in compliance with the American Association for Accreditation for Laboratory Animal Care at the Perinatal Research Center at the University of Colorado School of Medicine (Aurora, CO, USA).

### 2.2 Mouse model of fetal growth restriction

FGR was induced by maternal calorie restriction (CR) during the second half of gestation as previously described (14). Briefly, singly housed female C57BL/6 mice mated to C57BL/6 males were provided with ad lib access to standard laboratory chow (Teklad 2020X, Inotiv, Indianapolis, IN, USA) from gestational day E0-E9. Pregnant dams were randomized to continued ad lib chow (control, n = 7) or provision of chow diet to meet 70% of estimated calorie needs (CR, n = 13) from day E9 through delivery. After delivery, all dams had ad libitum access to standard laboratory chow throughout lactation. In this model, pups from CR dams weigh approximately 20% less at the end of gestation compared to controls, with catch-up weight gain achieved by day of life 2 (14). Only litters with at least 6 surviving pups were used for further study to minimize the effects of litter size on postnatal growth.

### 2.3 Outcome measures and feces collection

Dams were weighed on an electronic scale before mating and then daily throughout pregnancy. Gestational weight gain was defined as weight on day E18.5 minus weight immediately prior to mating (in grams). Litter size was determined by counting fetuses from a subset of dams euthanized on day E18.5 (n = 4 litters) or by counting pups on the morning after parturition (n = 15 litters). Pup weights were obtained on day of life 0 (n = 3 control litters, n = 4 FGR litters), day of life 1 (n = 6 control litters, n = 9 FGR litters), day of life 7 (n = 7 control litters, n = 8 FGR litters) and ages 3, 4 and 16 weeks. Pups were weaned on day of life 21 to a defined laboratory diet (7% calories from fat, Harlan TD.09283, Inotiv). Fresh fecal pellets were collected from pregnant dams on day E18.5 and from offspring on day of life 21 (n = 2 control litters, n = 3 FGR litters) and day of life 28 (n = 6 control litters, n = 7 FGR litters) by allowing each animal to walk around in a clean cage until one or more fecal pellets were produced. Pellets were frozen and stored at -80°C.

### 2.4 Fecal DNA isolation and microbiome sequencing

The fecal microbiome was assessed as previously described (14, 24). Briefly, bacterial DNA was extracted using a DNeasy PowerSoil HTP 96 kit (Qiagen, Redwood City, CA) including a bead-beating step on a TissueLyser II in 96-well PowerBead plates (Qiagen). Bacterial genomic DNA was used for amplification of the V4 variable region of the 16S rRNA gene using 515F/806R primers. Paired-end sequencing of pooled amplicons was accomplished on an Illumina MiSeq Instrument (Illumina, San Diego, CA).

### 2.5 Statistical analysis

Comparison groups were control versus CR dams, and control versus FGR offspring at two separate timepoints, 3 and 4 weeks of age. Statistical analyses and visualizations were performed in R version 4.2.2 or GraphPad Prism version 10.1.1. The fecal microbiome was analyzed as previously described (14, 24). Briefly, QIIME2 was used for data processing. Microbial counts, taxonomy information and sample metadata were imported using the *phyloseq* package (44). The *microbiome* package ‘core’ function was used to eliminate taxa that did not have at least 5 counts in 5% of samples. Following pre-processing, the following sample sizes were used in the final analysis; Dams: control n = 7 control, CR n = 13; age 3 weeks: control n = 14 (7 female), FGR n = 14 (6 female); age 4 weeks: control n = 15 (8 female); FGR n = 18 (9 female). Alpha diversity was determined using the *microeco* package (45) and Student’s t-test (for sex-combined analyses) or two-way ANOVA (for sex-stratified analyses) were used to test differences between groups. Beta diversity was assessed using Aitchison distance, Bray-Curtis dissimilarity, and Jaccard dissimilarity. Multidimensional scaling was used to visualize beta diversity for each group and statistical difference was tested using PERMANOVA with 999 permutations. Multivariable associations between maternal diet and taxonomic abundance were assessed using the *MaAsLin2* package (46). Maternal diet was considered a fixed effect and offspring analyses were adjusted for litter. Taxa were agglomerated at the genus level. All P-values were false discovery rate-adjusted (FDR; Benjamini–Hochberg, q-values) and features with q < 0.3 were considered significant (the default value for *MaAsLin2*). Findings passing an un-adjusted P < 0.05 are also included in results. The OTU relative abundance is visualized on a log-transformed axis in figures. Associations between microbial taxa and offspring outcomes were summarized using distance-based redundancy analysis (db-RDA) Bray–Curtis distances and further tested using Pearson correlations in *microeco* using the cal_cor function.

## 3 Results

### 3.1 Animal characteristics

Calorie restricted (CR) dams gained 17% less weight over the duration of gestation compared to control dams (p=0.037, **Table 1**). Litter size was slightly higher in CR dams (mean ± SD: 8.54 ± 0.97) compared to control (7.43 ± 1.40; p=0.050). We also confirmed fetal growth restriction (FGR) of the offspring of CR dams with lower weights on day of life 0 by 10% (control 1.31 ± 0.03g; FGR: 1.18 ± 0.06g; p = 0.025). From day of life 1, FGR and control offspring weights did not differ on day of life 1, 7, 21, or 28 (**Table 1**). FGR offspring used for the Week 4 microbiome dataset had slower weight gain from day 21 (week 3) to day 28 (week 4; p = 0.049). In addition, no significant differences were shown between control and FGR offspring for weight gain at 16 weeks of age (**Table 1**).

**Table 1:** Characteristics for dams and offspring at 3 and 4 weeks of age.

	Dams					
	Control (n=7)	CR (n=13)	P-value			
**Dam Weight Gain (g)**	14.72 ± 2.97	12.16 ± 2.12	**0.037**			
**Litter size (n)**	7.43 ± 1.40	8.54 ± 0.97	0.050			
**Day 0 Pup Weight (g)**	1.31 ± 0.03	1.18 ± 0.06	**0.025**			
**Day 1 Pup Weight (g)**	1.29 ± 0.07	1.25 ± 0.08	0.341			
**Day 7 Pup Weight (g)**	3.48 ± 0.30	3.59 ± 0.25	0.449			
	Offspring – Age 3 Weeks			Offspring – Age 4 Weeks		
	Control (n=14)	FGR (n=14)	P-value	Control (n=15)	FGR (n=18)	P-value
**Body Weight at 21 days (g)**	7.25 ± 1.23	9.35 ± 3.28	0.113	8.12 ± 0.56	8.28 ± 0.82	0.493
**Body Weight at 28 days (g)**	13.63 ± 1.00	13.56 ± 1.04	0.869	14.05 ± 1.17	13.63 ± 1.36	0.327
**Weight gain between 21 and 28 days (g)**	5.74 ± 0.67	5.30 ± 0.75	0.256	5.93 ± 0.82	5.34 ± 0.89	**0.049**
**Weight gain between 3 and 16 weeks (g)**	13.59 ± 0.85	13.80 ± 1.01	0.667	14.05 ± 1.17	13.63 ± 1.36	0.328

Values are presented as mean ± SD. P-values were determined by Student’s t-test. Bold signifies p < 0.05. CR = caloric restriction; FGR = fetal growth restriction.

### 3.2 Caloric restriction during pregnancy impacts maternal fecal microbiome composition

**Figure 1:**
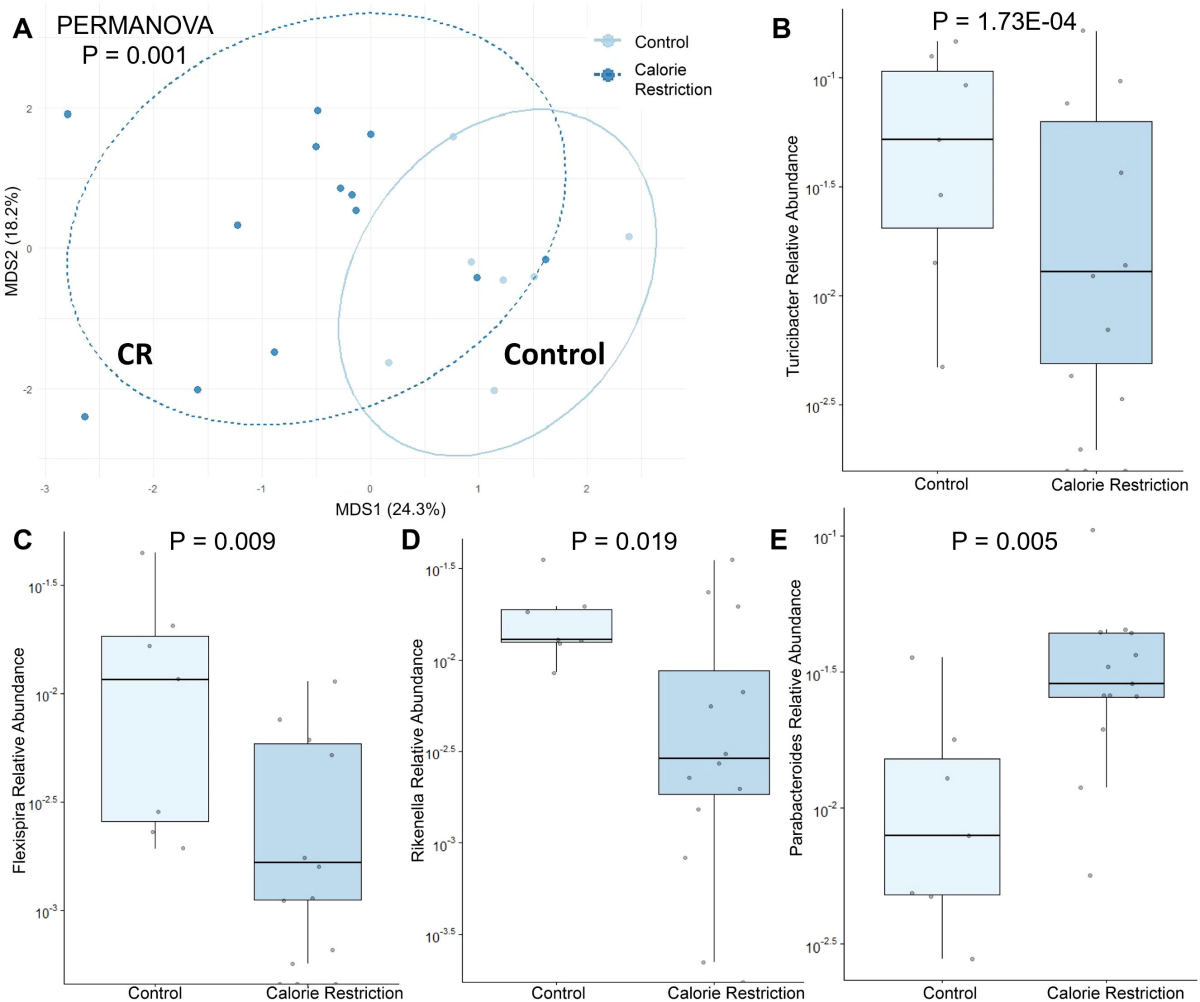
Fecal microbiome differences in control and calorie restricted dams at day E18.5. **(A)** Aitchison distance in control and calorie restricted dams. Relative abundance of **(B)** *Turicibacter* **(C)** *Flexispira* **(D)** *Rikenella* and **(E)** *Parabacteroides* in control and FGR dams. CR, caloric restriction.

The fecal microbiome from CR dams (n = 13) at gestational day E18.5 was compared to that from control dams (n = 7). We saw no statistical difference for any measure of alpha diversity including Chao1, Shannon, Simpson or Fisher indices (**Table 2**). Global genus-level composition (beta diversity) differed between CR and control dams by all three distance measures tested (**Table 3**): Aitchison (**Figure 1A**), Bray-Curtis, and Jaccard. At the genus level, we found decreased relative abundance of *Turicibacter* (**Figure 1B**), *Flexispira* (**Figure 1C**), and *Rikenella* (**Figure 1D**), and increased relative abundance of *Parabacteroides* (**Figure 1E**) and *Prevotella* in CR dams (all p<0.05, **Table 3**). CR dams also had lower relative abundance of *Bifidobacterium* based on q value of < 0.03 (q = 0.29, p = 0.061; **Table 4**).

**Table 2:** Alpha diversity. Values are presented as mean ± SD. P-values were determined by Student’s t-test.

	Dams			Offspring – Age 3 weeks			Offspring – Age 4 Weeks		
	Control (n=7)	CR (n=13)	P-value	Control (n=14)	FGR (n=14)	P-value	Control (n=15)	FGR (n=18)	P-value
Chao1	28.14 ± 3.19	28.08 ± 3.95	0.970	21.00 ± 6.56	23.14 ± 3.11	0.280	27.60 ± 2.41	26.94 ± 2.67	0.469
Shannon	2.20 ± 0.30	2.08 ± 0.33	0.439	1.68 ± 0.30	1.94 ± 0.21	**0.016**	2.08 ± 0.23	1.93 ± 0.22	0.063
Simpson	0.81 ± 0.08	0.79 ± 0.11	0.671	0.70 ± 0.10	0.79 ± 0.06	**0.009**	0.81 ± 0.06	0.78 ± 0.05	0.177
Fisher	3.18 ± 0.23	3.04 ± 0.43	0.427	n/a	n/a		2.84 ± 0.24	2.79 ± 0.27	0.551

Bold signifies p < 0.05. CR = caloric restriction; FGR = fetal growth restriction.

**Table 3:** Beta diversity.

	Dams	Offspring – Age 3 Weeks			Offspring – Age 4 Weeks		
		All	Males	Females	All	Males	Females
Jaccard	**0.009**	**0.001**	**0.001**	**0.002**	0.237	0.603	0.429
Bray-Curtis	**0.007**	**0.001**	0.078	**0.002**	0.316	0.623	0.638
Aitchison	**0.001**	**0.001**	**0.036**	**0.008**	**0.034**	0.139	0.129

P-values for the effect of maternal diet on the fecal microbiome using different measures of beta diversity in dams and offspring. Bold = p < 0.05.

**Table 4:** Fecal microbiome from calorie restricted dams compared to control dams at day E18.5.

Phylum	Class	Order	Family	Genus	Coefficient	Std Dev	P value	Q value	# not zero
Decreased Abundance									
Firmicutes	Bacilli	Turicibacterales	Turicibacteraceae	Turicibacter	-4.623	0.981	**1.73E-04**	**0.006**	18
Proteobacteria	Epsilonproteobacteria	Campylobacterales	Helicobacteraceae	Flexispira	-2.525	0.864	**0.009**	**0.109**	17
Bacteroidetes	Bacteroidia	Bacteroidales	Rikenellaceae	Rikenella	-2.462	0.957	**0.019**	**0.172**	19
Actinobacteria	Actinobacteria	Bifidobacteriales	Bifidobacteriaceae	Bifidobacterium	-2.233	1.116	0.061	**0.290**	17
Increased Abundance									
Bacteroidetes	Bacteroidia	Bacteroidales	Porphyromonadaceae	Parabacteroides	1.681	0.520	**0.005**	**0.083**	20
Bacteroidetes	Bacteroidia	Bacteroidales	Paraprevotellaceae	Prevotella	3.124	1.298	**0.027**	**0.195**	19
Bacteroidetes	Bacteroidia	Bacteroidales	Prevotellaceae	Prevotella	1.047	0.458	**0.035**	**0.208**	20
Bacteroidetes	Bacteroidia	Bacteroidales	Bacteroidaceae	Bacteroides	1.416	0.730	0.068	**0.290**	20

(n = 20 total).

Bold signifies p < 0.05.

### 3.3 Association of maternal bacterial taxa with pregnancy and early offspring outcomes

**Figure 2:**
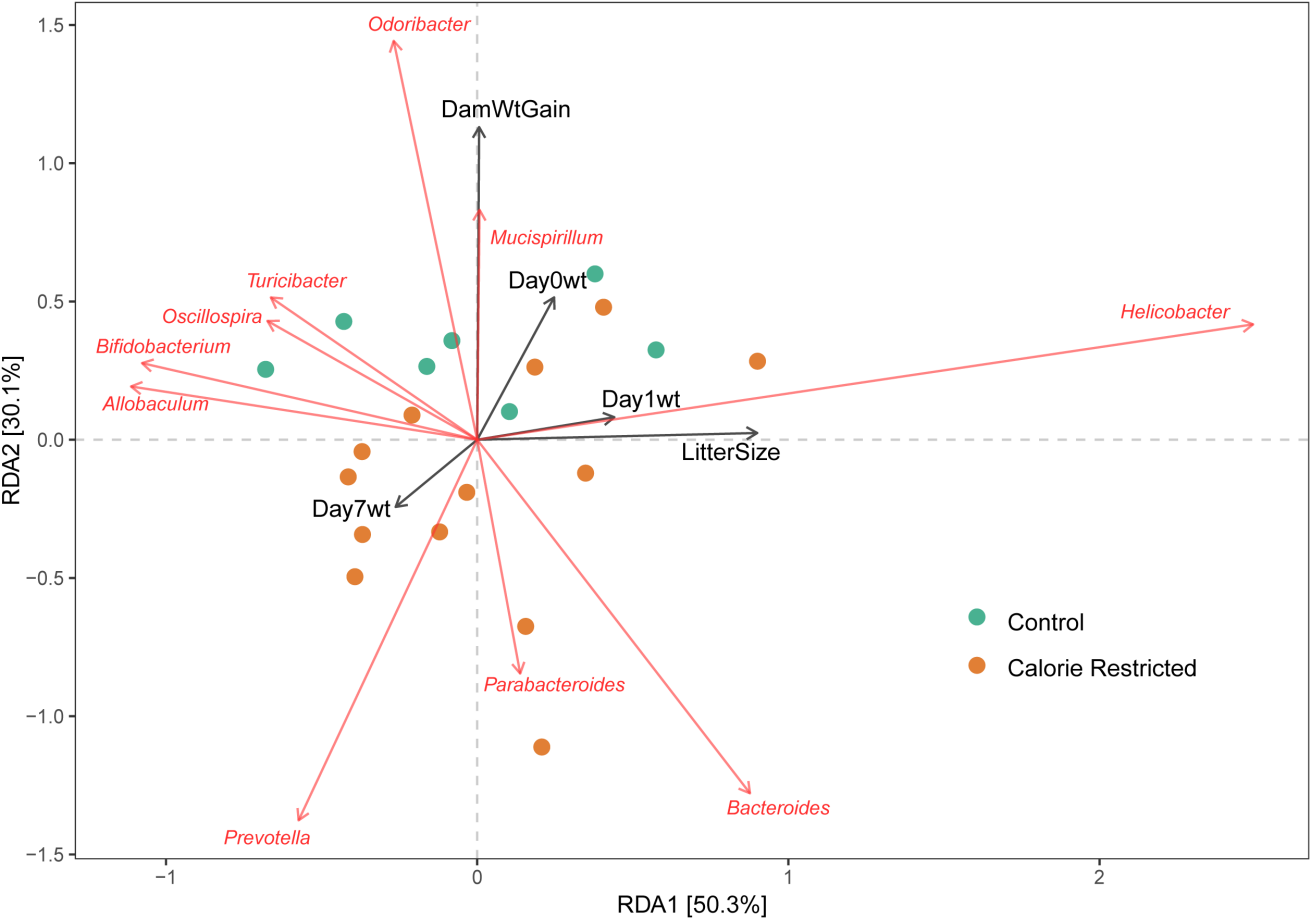
Association of maternal taxa with pregnancy and offspring outcomes. Distance-based redundancy analysis for genus-level taxa with selected outcomes. DamWtGain = Weight gain across pregnancy; Day0Wt, Average pup weight on day of life 0; Day1Wt, Average pup weight on day of life 1; Day7Wt, Average pup weight on day of life 7; LitterSize, Number of pups per litter.

We used dbRDA to test for associations between maternal fecal microbial taxa and outcomes of pregnancy (maternal weight gain, litter size) and offspring (average pup weight on day of life 0, 1 and 7). Weight gain during pregnancy showed positive alignment with relative abundance of *Mucispirillum* and *Odoribacter*, and negatively alignment with *Parabacteroides* (**Figure 2**). The number of pups per litter and average pup weight on day of life 1 were positively aligned with *Helicobacter* and negatively aligned with *Allobaculum* and *Bifidobacterium* abundance. Using Pearson correlation and nominal p-values, several taxa were negatively associated with weight of offspring from CR dams including *Bifidobacterium*, *Blautia*, and *Coprococcus* (**Supplementary Figure S1**). In control dams, day 1 offspring weight was negatively associated with relative abundance of *Roseburia*, *Ruminococcus*, and *Flexispira*. No maternal taxa were associated with offspring weight at day 7 of life in either CR or control dams. After FDR-correction, the only remaining significant correlation was a negative association between *Alistipes* abundance in control dams and Day 0 offspring weight.

### 3.4 Impact of fetal growth restriction due to maternal calorie restriction on offspring fecal microbiome composition at age 3 weeks

**Figure 3:**
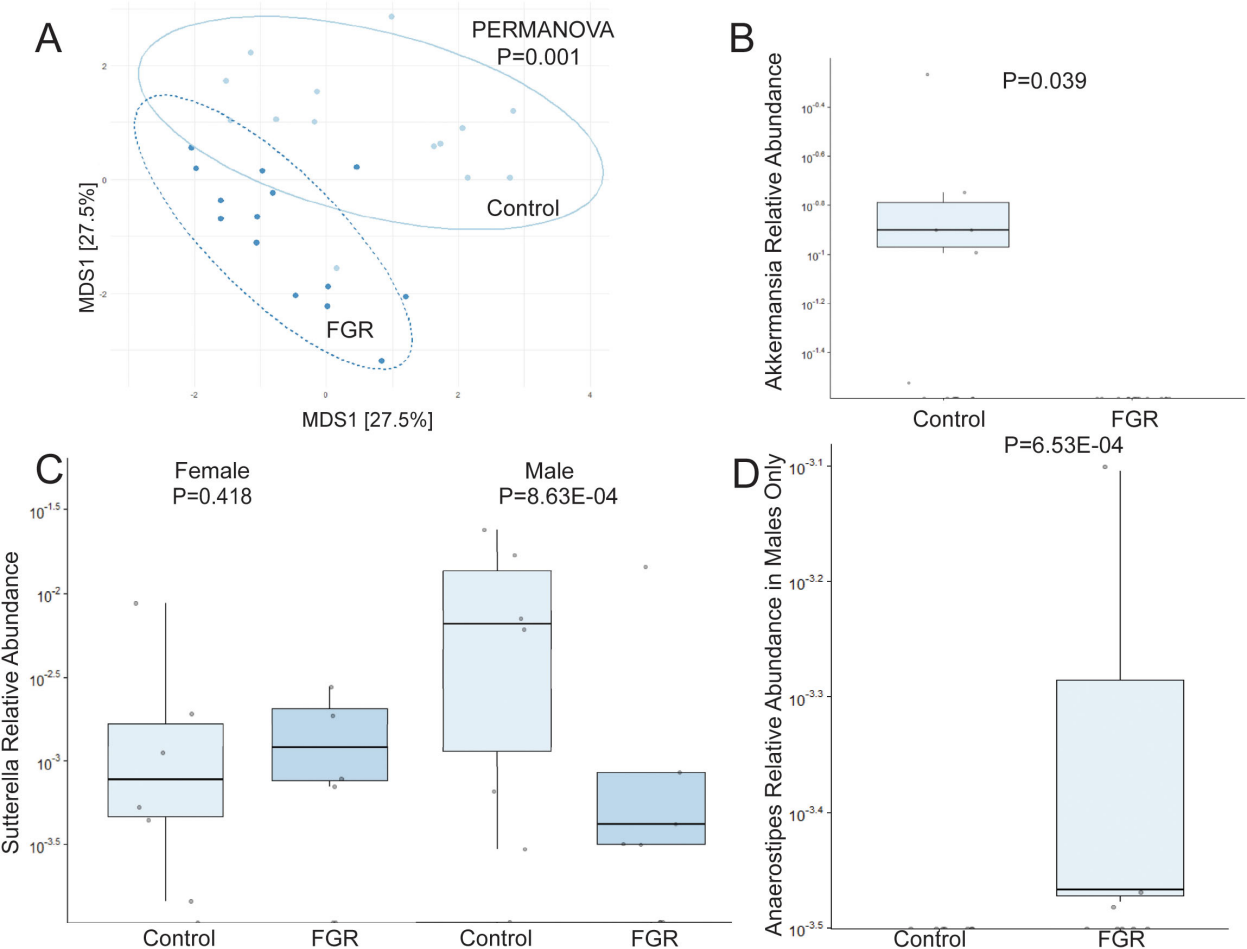
Fecal microbiome differences in control and fetal growth restricted offspring at age 3 weeks. **(A)** Aitchison distance in control and fetal growth restricted offspring at age 3 weeks. Relative abundance of **(B)** *Akkermansia* **(C)** *Sutterella* by offspring sex, and **(D)** *Anaerostipes* in control and FGR offspring. FGR, fetal growth restriction.

Analysis of offspring fecal microbiome, regardless of sex, demonstrated no differences in any measure of alpha diversity were detected between control (n = 14) and FGR (n = 14) at 3 weeks of life (**Table 2**). Beta diversity significantly differed between control and FGR offspring by Aitchison (**Figure 3A**), Bray-Curtis and Jaccard (**Table 3**). At the genus level, FGR offspring had decreased relative abundance of *Akkermansia* (**Figure 3B**) and *Sutterella* (**Figure 3C**) and increased relative abundance of *Anaerostipes* (**Figure 3D**) and *Paraprevotella* (**Table 5**).

**Table 5:** Fecal microbiome from fetal growth restricted offspring compared to control offspring at 3 weeks of age.

Phylum	Class	Order	Family	Genus	Coefficient	Std Dev	P value	Q value	# not zero
Decreased Abundance									
Proteobacteria	Betaproteobacteria	Burkholderiales	Alcaligenaceae	Sutterella	-7.289	2.592	**0.014**	0.332	21
Verrucomicrobia	Verrucomicrobiae	Verrucomicrobiales	Verrucomicrobiaceae	Akkermansia	-3.326	1.458	**0.039**	0.542	6
Increased Abundance									
Firmicutes	Clostridia	Clostridiales	Lachnospiraceae	Anaerostipes	2.240	0.272	**9.90E-07**	**4.92E-05**	4
Bacteroidetes	Bacteroidia	Bacteroidales	Paraprevotellaceae	Paraprevotella	7.693	3.146	**0.028**	0.441	18

(n = 28 total).

Bold signifies p < 0.05.

We performed additional analyses to test for sex-specific differences between males (n = 7 control, n = 8 FGR) and females (n = 7 control, n = 6 FGR). For alpha diversity, two-way ANOVA revealed a significant association between intrauterine growth and microbial diversity (Shannon diversity index p = 0.021, Simpson index p = 0.022) but not richness (Chao1 p = 0.25). There was no significant interaction between sex and growth (all p > 0.1). Beta diversity differed between control and FGR male offspring when assessed by Aitchison and Jaccard distances but not by Bray-Curtis (**Table 3**). Beta diversity between control and FGR females differed according to all assessment methods (**Table 3**). Compared to control males, FGR males had increased relative abundance of *Anaerostipes* and *Paraprevotella* and decreased relative abundance of *Sutterella* (**Figure 3C**; **Supplementary Table S1**). FGR females had higher relative abundance of *Oscillospira* and *Alistipes* compared to control females (**Supplementary Table S1**).

### 3.5 Impact of fetal growth restriction due to maternal calorie restriction on offspring fecal microbiome composition at age 4 weeks

**Figure 4:**
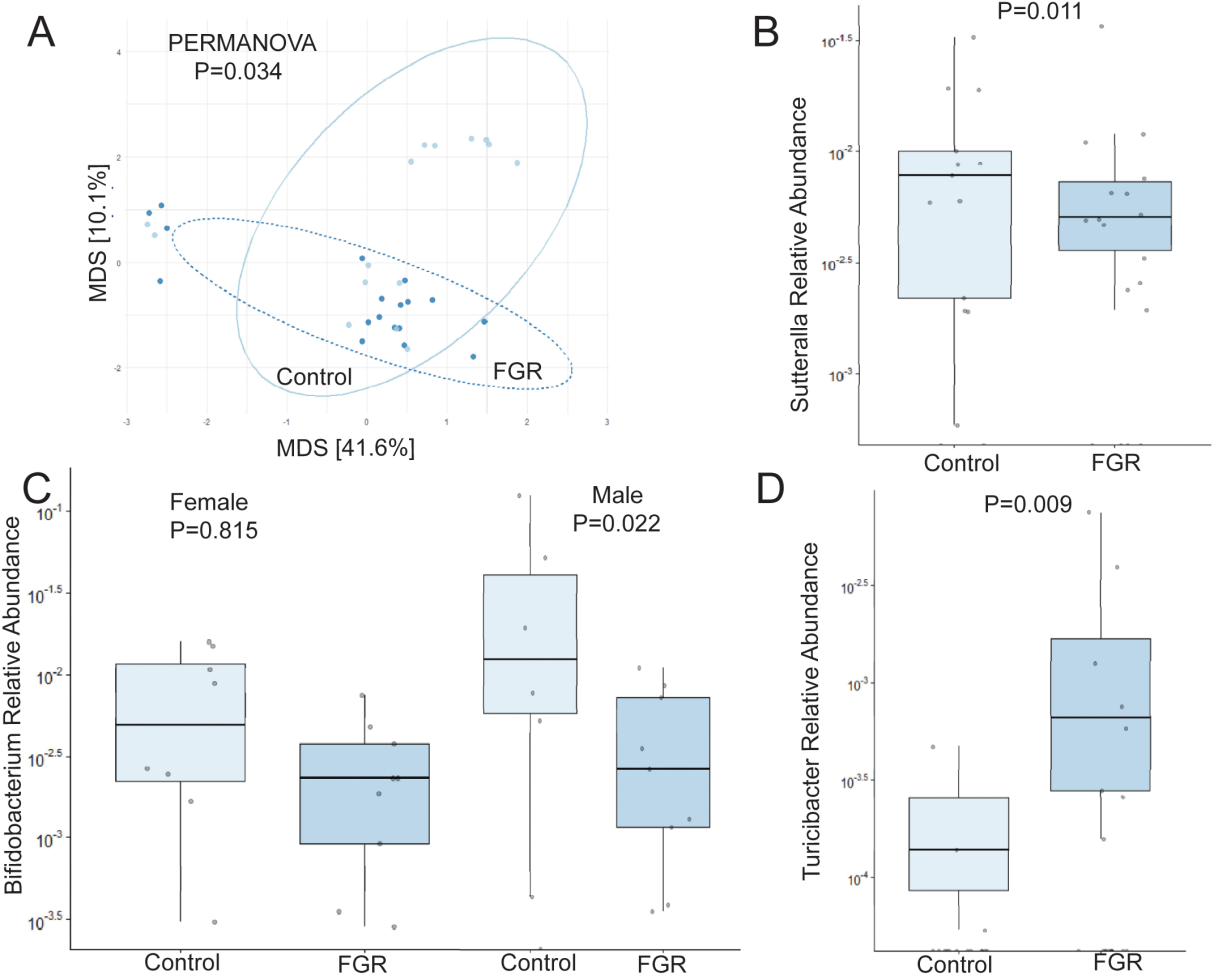
Fecal microbiome differences in control and fetal growth restricted offspring at age 4 weeks. **(A)** Aitchison distance in control and fetal growth restricted offspring at age 4 weeks. Relative abundance of **(B)** *Sutterella* **(C)** *Bifidobacterium* by offspring sex, and **(D)** *Turicibacter* in control and FGR offspring. FGR, fetal growth restriction.

Analysis of offspring fecal microbiome, regardless of sex, demonstrated no differences in alpha diversity were detected between control (n = 15) and FGR (n = 18) at 4 weeks of life (**Table 2**). Beta diversity significantly differed between control and FGR offspring by Aitchison only (**Figure 4A**; **Table 3**). At the genus level, FGR animals had decreased relative abundance of *Allobaculum*, *Sutterella* (**Figure 4B**), *Bifidobacterium* (**Figure 4C**), *Lactobacillus* and among others and increased relative abundance of *Turcibacter* (**Figure 4D**), *Dorea*, and *Roseburia* (**Table 6**).

**Table 6:** Fecal microbiome from fetal growth restricted offspring compared to control offspring at 4 weeks of age.

Phylum	Class	Order	Family	Genus	Coefficient	Std Dev	P value	Q value	# not zero
Decreased Abundance									
Firmicutes	Erysipelotrichi	Erysipelotrichales	Erysipelotrichaceae	Allobaculum	-10.824	0.871	**1.23E-09**	**1.22E-07**	29
Bacteroidetes	Bacteroidia	Bacteroidales	Porphyromonadaceae	Parabacteroides	-5.575	0.914	**1.54E-05**	**3.64E-04**	33
Firmicutes	Bacilli	Lactobacillales	Lactobacillaceae	Lactobacillus	-4.951	0.922	**6.27E-05**	**0.001**	33
Bacteroidetes	Bacteroidia	Bacteroidales	Paraprevotellaceae	Paraprevotella	-6.288	1.531	**0.001**	**0.012**	27
Actinobacteria	Actinobacteria	Bifidobacteriales	Bifidobacteriaceae	Bifidobacterium	-5.026	1.510	**0.004**	**0.043**	32
Bacteroidetes	Bacteroidia	Bacteroidales	Rikenellaceae	AF12	-4.259	1.355	**0.006**	**0.057**	32
Proteobacteria	Betaproteobacteria	Burkholderiales	Alcaligenaceae	Sutterella	-3.905	1.356	**0.011**	**0.079**	27
Bacteroidetes	Bacteroidia	Bacteroidales	Rikenellaceae	Alistipes	-2.858	1.476	0.071	**0.281**	28
Increased Abundance									
Firmicutes	Bacilli	Turicibacterales	Turicibacteraceae	Turicibacter	4.459	1.496	**0.009**	**0.070**	11
Firmicutes	Clostridia	Clostridiales	Lachnospiraceae	Dorea	4.084	1.430	**0.011**	**0.080**	16
Firmicutes	Clostridia	Clostridiales	Lachnospiraceae	Roseburia	4.429	1.693	**0.019**	**0.109**	10

(n = 33 total).

Bold signifies p < 0.05.

As above, we tested for sex-specific differences between males (n = 7 control, n = 9 FGR) and females (n = 8 control, n = 9 FGR). For alpha diversity, two-way ANOVA revealed no significant associations between intrauterine growth and microbial diversity, nor any interaction between sex and growth (all p > 0.05). Beta diversity also did not differ between FGR and controls for males or females (**Table 2**). At the genus level, male FGR offspring had decreased relative abundance of *Allobaculum*, *Bifidobacterium* (**Figure 4C**), *Lactobacillus* and *Alistipes* among others and increased relative abundance of *Dorea* and *Roseburia* (**Supplementary Table S2**). FGR females had lower relative abundance of *Rikenella*, *Akkermansia*, and *Bilophilia*, and increased abundance of *Dorea* and *Lactobacillus* (**Supplementary Table S2**).

### 3.6 Associations between offspring weight and bacterial taxa at ages 3 and 4 weeks

**Figure 5:**
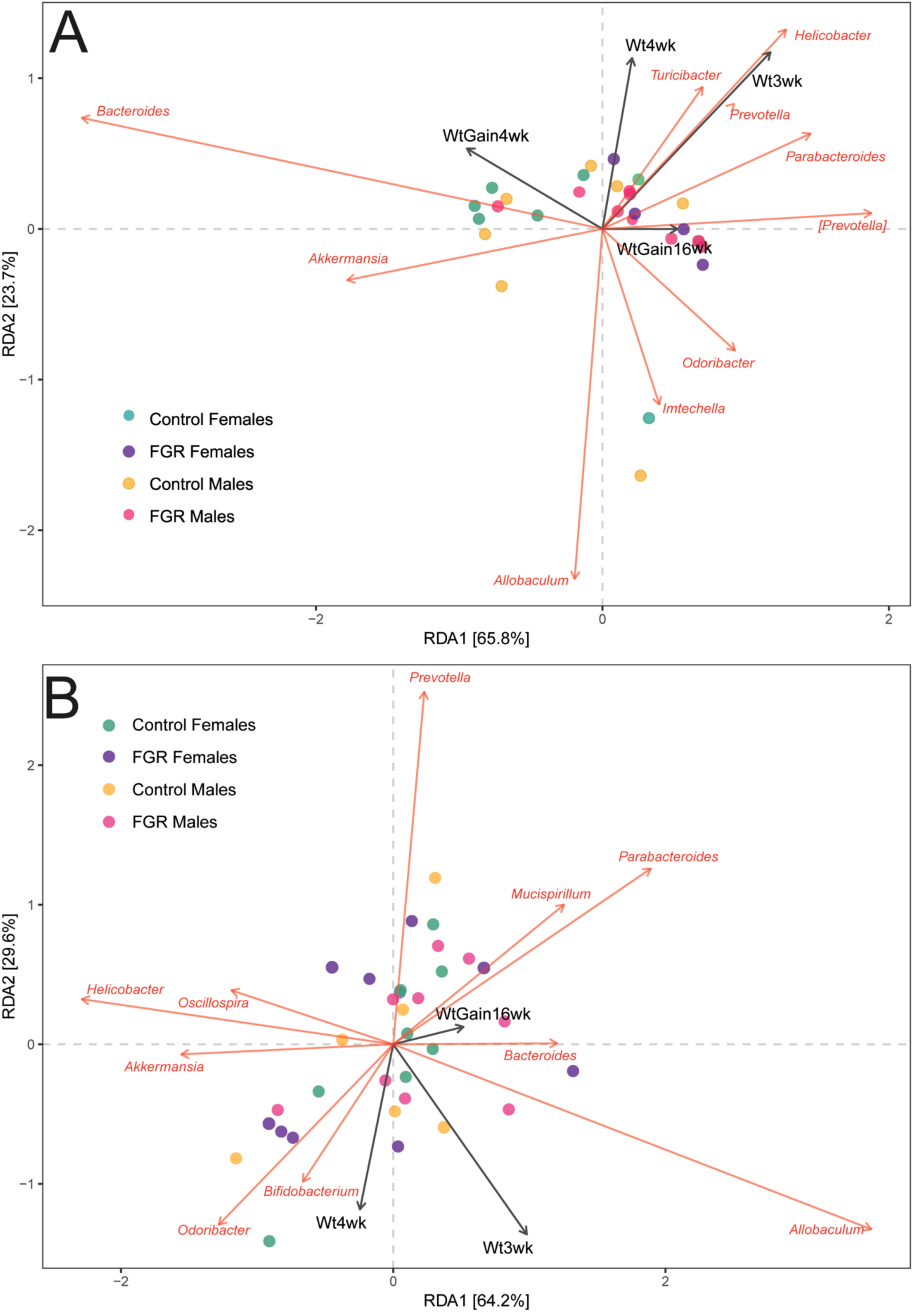
Association of offspring microbial abundance with weight outcomes. Distance-based redundancy analysis for offspring genus-level taxa with selected outcomes. **(A)** Microbiome at age 3 weeks; **(B)** Microbiome at age 4 weeks. Wt3wk, weight at age 3 weeks; Wt4wk, weight at age 4 weeks; WtGain4wk, weight gain from age 3 to 4 weeks; WtGain16wk, weight gain from age 3 to 16 weeks.

We tested for associations between fecal microbial taxa and offspring weight at 21 and 28 days, and 16 weeks of life as well as weight gain between 21 and 28 days of life. At age 3 weeks, weight was aligned with *Helicobacter, Turcibacter*, and *Prevotella* abundances and weight at 4 weeks was negatively aligned with *Allobaculum* abundance. Weight at age 16 weeks was negatively aligned with *Akkermansia* abundance both age 3 weeks (**Figure 5A**) and age 4 weeks (**Figure 5B**).

Using Pearson correlations, negative associations were noted in control animals after between several taxa and weight at age 3 weeks including *Bifidobacterium*, *Dorea*, *Aldercreutzia*, and *Anaeroplasma* (**Supplementary Figure S2**). In FGR animals, a positive association was noted between abundance of *Dorea* and *Coprococcus* with weight at age 3 weeks (**Supplementary Figure S2**, top panel) but did not remain significant after FDR correction (**Supplementary Figure S2**, bottom panel). There was no significant association with taxa at 3 weeks and 16-week weight. At age 4 weeks, no associations between microbial taxa and weight outcomes passed FDR correction in either FGR or control animals. With nominal p-values, we noted a positive association between body weight at 4 weeks and abundance of *Bifidobacterium*, *Flexispira* and *Lactobacillus*, and a negative association with *Parabacteroides* abundance for control animals only (**Supplementary Figure S3**). There were no significant associations in FGR animals, nor did we identify any significant association between bacterial taxa at 4 weeks and 16-week weight.

We also performed sex-stratified analyses at age 4 weeks. In males, no associations between microbial taxa and weight outcomes passed FDR correction in either FGR or control animals. With nominal p-values, we noted a positive association between *Bifidobacterium* and *Flexispira* abundances and body weight at age 4 weeks and weight gain between weeks 3 and 4, and negative association between *Paraprevotella* and *Ruminococcus* abundance and weight gain between weeks 3 and 4 only in control offspring (**Supplementary Figure S4**). In females, we similarly saw no associations between the microbiome and growth in FGR offspring with FDR correction (**Supplementary Figure S5**, bottom panel). With nominal p-values, we noted a negative association between relative abundance of *Akkermansia* and *Parabacteroides* and weight gain from 3 to 4 weeks, and a positive association between *Blautia* abundance and weight gain from 3 to 16 weeks (**Supplementary Figure S5**, top panel). In control females, we noted positive associations between body weight at 4 weeks with multiple taxa including *Aldercreutzia*, *Coprococcus*, *Dorea*, *Roseburia*, and *Ruminococcus* (**Supplementary Figure S5**).

## 4 Discussion

The ability to prevent fetal growth restriction and/or mitigate its adverse effects would impact the health of millions of children worldwide (3, 8, 47). Gut microbial dysbiosis is being increasingly recognized as a contributing mechanism to adverse pregnancy outcomes, including FGR (15, 48). Here, we used a mouse model of undernutrition to test for differences in maternal and offspring fecal microbial ecology and associations with pregnancy and offspring outcomes. Our findings indicate that calorie restriction during pregnancy impacts maternal gut microbiome with modest associations between microbial taxa and pregnancy outcomes that differed between control and calorie-restricted animals. Fecal microbiome showed further differences in offspring at two different time points but minimal associations with postnatal growth.

The present study builds upon our prior work demonstrating fecal microbial dysbiosis in adult FGR offspring (14). One mechanism underlying offspring dysbiosis could be inheritance of an abnormal microbiome during and after birth (48). It is important to note that our study was not designed to assess vertical transmission of the microbiome. Multiple studies have demonstrated similarities between parental and offspring fecal microbiota in humans (49) and mice (50). However, offspring microbiome in early developmental stages (weaning and immediately postweaning) is quite different than the adult microbiome. Further, coprophagia among mice results in rapid sharing of fecal microbes (51, 52), emphasizing the appropriateness of our study design. Future work could perform paired analyses of dams and their offspring and/or use metagenomic sequencing to make species and strain level comparisons.

Calorie-restricted dams had notable reduction in Turicibacter, a bacterium previously shown to modulate host bile acid and lipid metabolism (53) and to be associated with beneficial short-chain fatty acids (SCFAs) (54) and reduction of inflammation (55, 56). Our results are similar to previous studies which also showed lower Turicibacter abundance in the setting of stress during pregnancy, including heat stress in pigs (57) and HIV infection in humans (58). Turicibacter abundance is also reduced in mouse models of diet-induced obesity (59), colitis (56), and some gastrointestinal cancers (55). Although prior work demonstrated that Turicibacter is highly heritable (60), FGR offspring in this study had increased Turicibacter abundance at age 4 weeks, particularly among males. We did not observe significant differences in Turicibacter abundance at 3 weeks, nor at 16 weeks as previously published (14). Similarly, we previously described lower Turicibacter abundance in 1-month-old infants born to mothers with obesity (24) but not among the mothers during pregnancy at any timepoint (34). Because different strains may influence the role of this taxon (53), additional work is needed to determine the specific species and/or strains present and whether this differs between dams and offspring.

We identified a decrease in Rikenella during pregnancy in CR dams compared to control and in FGR female offspring at 4 weeks. Rikenella species have previously been shown to be decreased in piglets with FGR (61), mice with chronic stress (62) and diabetes (63), humans with obesity (64, 65), and infants born to mothers with gestational diabetes (42). Alistipes, also in the Rikenellaceae family, was decreased in FGR males at age 4 weeks, without detectable differences in dams. In contrast, maternal Alistipes abundance was decreased in human pregnancies impacted by FGR (15) and gestational diabetes (66) and may be associated with impaired glucose homeostasis in pregnancies impacted by overweight (67). The role of Alistipes in health is still being clarified and may depend not only on the species, but also on the specific disease being studied, possibly due to differential production of SCFAs (68). Rikenella and Alistipes were recently found to be higher in female mice exposed to heat stress to induce FGR (69). Therefore, the varying etiologies for FGR may differentially impact the maternal and offspring fecal microbiome and warrants further exploration.

Parabacteroides and Prevotella were enriched in CR dams at the end of pregnancy. Both taxa are important for carbohydrate degradation and are also enriched in women with obesity and gestational diabetes (70–74). Our findings are not consistent with those from humans showing an association between maternal and infant Parabacteroides abundance (72). In our model, 4-week-old male FGR offspring had reduced abundance of both Prevotella and Parabacteroides compared to controls, while in adulthood FGR females had increased Prevotella abundance only when challenged with a high fat diet (14). These findings should be viewed in light of a dynamic microbiome in the early stages of the post-weaning period. Our results highlight the complexity of fecal microbial establishment during infancy.

A notable finding from our prior work was depletion of the beneficial bacterium Akkermansia in adult FGR males (14). In the present work, we report decreased Akkermansia in FGR offspring at age 3 weeks. At that timepoint, 6 of 14 control offspring (3 male, 3 female) had detectable Akkermansia compared to 0 of 14 FGR offspring. There were no differences in CR dams compared to controls, suggesting against impaired vertical transmission as the mechanism for diminished abundance. Akkermansia has been showed to be reduced in pregnancies impacted by pre-eclampsia (39, 75) and gestational diabetes mellitus (76), both associated with adverse fetal weight outcomes. We also demonstrate negative alignment of Akkermansia abundance at ages 3 and 4 weeks with adult weight. Studies in both mice and humans have demonstrated a beneficial role of Akkermansia on insulin sensitivity and excess adiposity (77). Our results suggest that the role of Akkermansia in metabolic health may begin early in the lifecourse.

FGR offspring also had decreased relative abundance of Sutterella at both ages 3 and 4 weeks. In contrast, at age 16 weeks, female FGR offspring had increased relative abundance of Sutterella without difference among males (14). It is currently unclear whether the health impacts of Sutterella change across the lifespan. A recent report showed a positive association between Sutterella, specifically S. wadsworthensis, with obesity in children and adolescents (78). Some Sutterella species appear to have modest pro-inflammatory effects and may have a role in immune system modulation (79). Despite high reported heritability of Sutterella in humans based on maternal fecal samples (49), we did not detect statistical differences in dams at the end of pregnancy.

There is emerging evidence that maternal gut bacteria impact feto-placental development including offspring body weight in both animal models and humans (32, 33, 37, 38). Therefore, we examined if maternal or offspring taxa were associated with maternal gestational weight gain, fetal weight, and offspring weight. Our results revealed associations between several maternal taxa and offspring weight early in infancy, particularly a negative association with Bifidobacterium abundance. Our results contrast with a prior study showing benefit of Bifidobacterium supplementation on fetal weight gain in germ-free mice (32). One study in humans did not find any specific association between Bifidobacterium species and birth weight (38). The discrepancies could be related to the different conditions of pregnancy or potentially to different species of Bifidobacterium (80). At age 4 weeks we noted a positive association between body weight and Bifidobacterium abundance in offspring. Work in human infants showed a negative association of early life Bifidobacterium abundance with rapid infancy weight gain (26) while supplementation of malnourished infants with B. infantis resulted in faster weight gain (81). It is possible that Bifidobacterium species support a healthy weight trajectory with precise effects varying by the larger environmental context.

As with all research, the present study has some limitations. There are multiple other mouse models of FGR including low protein diet without calorie restriction (82), heat stress (69), hypoxia (83), uterine artery ligation (84, 85), administration of thromboxane A2 analogs (86), and genetic manipulation (87). Whether the results presented here predict changes in other pre-clinical models of FGR is unknown. However, our results are relevant in part because maternal malnutrition is one of the most common causes of FGR worldwide (8, 88). In addition, calorie restriction produces a more severe phenotype compared to many other models (14). Our ability to detect sex differences in offspring may have been limited by statistical power due to necessary adjustment for co-housing, especially at age 3 weeks. Reduced housing density after weaning may strengthen our findings in future studies but the logistics of such a study may be moderated by animal welfare concerns (52). Compared to other models of FGR, we do not see a prolonged postnatal impact on offspring growth and weight, as in our model offspring complete catch up growth by day 2 (14). We suspect this is due to adequate and possibly increased consumption of milk in the first two days of life compared to control animals, but this has not been tested. The similarity in offspring body weight at age 21 and 28 days could explain the absence of significant associations with taxa and offspring weight/gain.

In conclusion, we demonstrate gut microbial dysbiosis in pregnant dams and offspring at two timepoints following maternal calorie restriction. We also report associations between specific bacterial taxa and offspring fetal and postnatal growth. Combined with results from others, the results presented here may suggest a functional role of the microbiome in mediating the relationship between maternal malnutrition during pregnancy and adverse growth outcomes in offspring. Interventions targeting the microbiome either during pregnancy or in young offspring may help optimize growth and improve health.

## Data Availability

The original contributions presented in the study are publicly available. This data can be found in the Sequence Read Archive database (https://www.ncbi.nlm.nih.gov/sra) with accession number PRJNA1162649.

## Supplementary material

Supplementary Figure S1 Association of maternal genus-level microbial abundance with pregnancy and offspring outcomes without false discovery rate adjustment. Red-blue spectrum signifies positive to negative correlations, respectively. P values determined by Pearson correlations. *p < 0.05, **p < 0.01, ***p < 0.001, BW, body weight; wt, weight.

Supplementary Figure S2 Association of offspring genus-level microbial abundance at 3 weeks with weight outcomes. Results are shown without **(top)** and with **(bottom)** False Discovery Rate Adjustment. Red-blue spectrum signifies positive to negative correlations, respectively. P values determined by Pearson correlations. *p < 0.05, **p < 0.01, ***p < 0.001, BW, body weight; wt, weight.

Supplementary Figure S3 Association of offspring genus-level microbial abundance at 4 weeks with weight outcomes without false discovery rate adjustment. Red-blue spectrum signifies positive to negative correlations, respectively. P values determined by Pearson correlations. *p < 0.05, **p < 0.01, ***p < 0.001, BW, body weight; wk, week; wt, weight.

Supplementary Figure S4 Association of male offspring genus-level microbial abundance at 4 weeks with weight outcomes without false discovery rate adjustment. Red-blue spectrum signifies positive to negative correlations, respectively. P values determined by Pearson correlations. *p < 0.05, **p < 0.01, ***p < 0.001, BW, body weight.

Supplementary Figure S5 Association of female offspring genus-level microbial abundance at 4 weeks with weight outcomes. Results are shown without (top) and with (bottom) False Discovery Rate Adjustment. Red-blue spectrum signifies positive to negative correlations, respectively. P values determined by Pearson correlations. *p < 0.05, **p < 0.01, ***p < 0.001, BW, body weight; wk, week; wt, weight.
