# Supplementary figures and images for: Calorie restriction during gestation impacts maternal and offspring fecal microbiome in mice
Source: Front Endocrinol (Lausanne). 2024 Oct 4;15:1423464. doi: 10.3389/fendo.2024.1423464 (PMC11487197; doi:10.3389/fendo.2024.1423464)

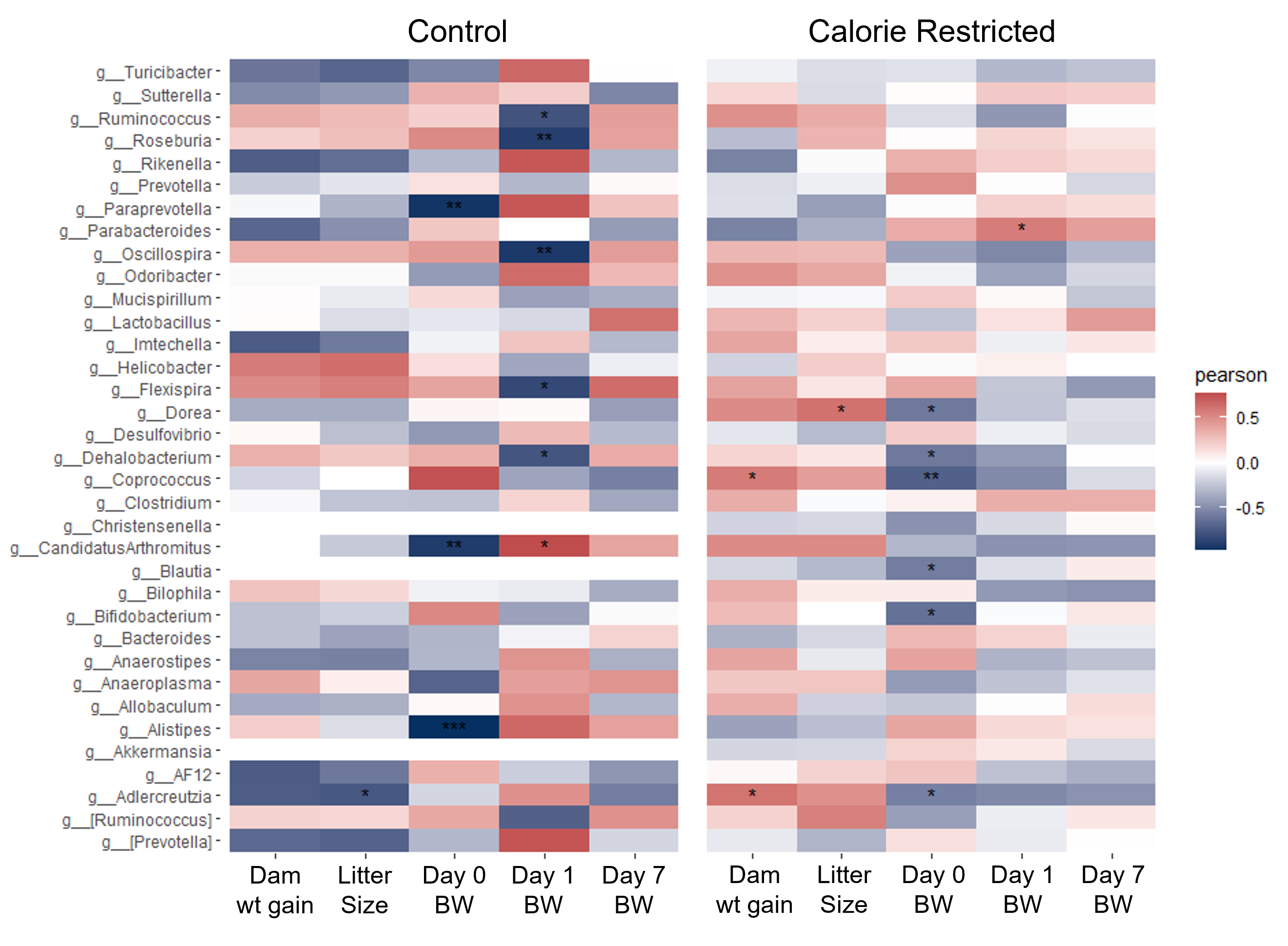

Supplement: Supplementary Figure S1 — Association of maternal genus-level microbial abundance with pregnancy and offspring outcomes without false discovery rate adjustment. Red-blue spectrum signifies positive to negative correlations, respectively. P values determined by Pearson correlations. *p < 0.05, **p < 0.01, ***p < 0.001, BW, body weight; wt, weight. [file Image1.tif]

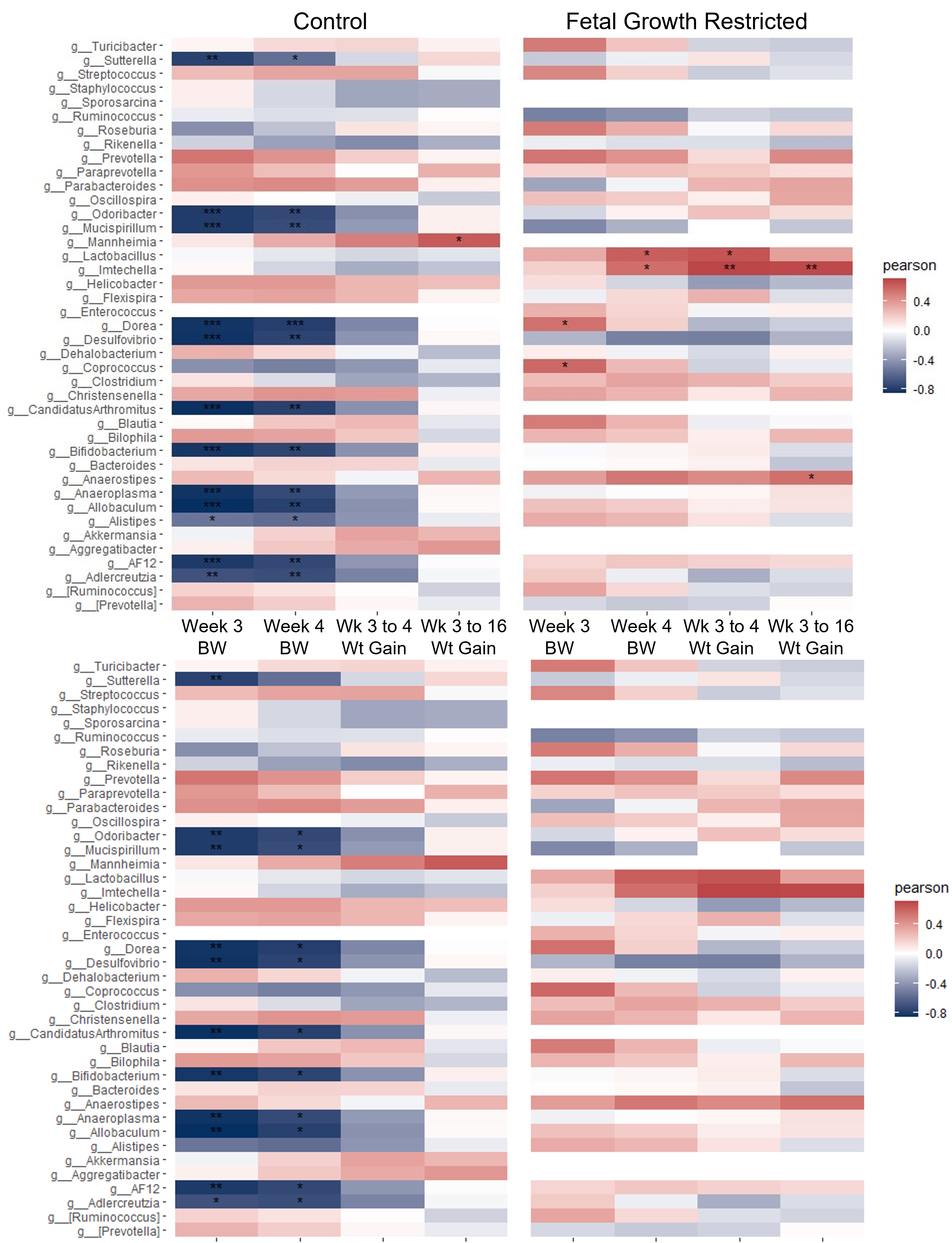

Supplement: Supplementary Figure S2 — Association of offspring genus-level microbial abundance at 3 weeks with weight outcomes. Results are shown without (top) and with (bottom) False Discovery Rate Adjustment. Red-blue spectrum signifies positive to negative correlations, respectively. P values determined by Pearson correlations. *p < 0.05, **p < 0.01, ***p < 0.001, BW, body weight; wt, weight. [file Image2.tif]

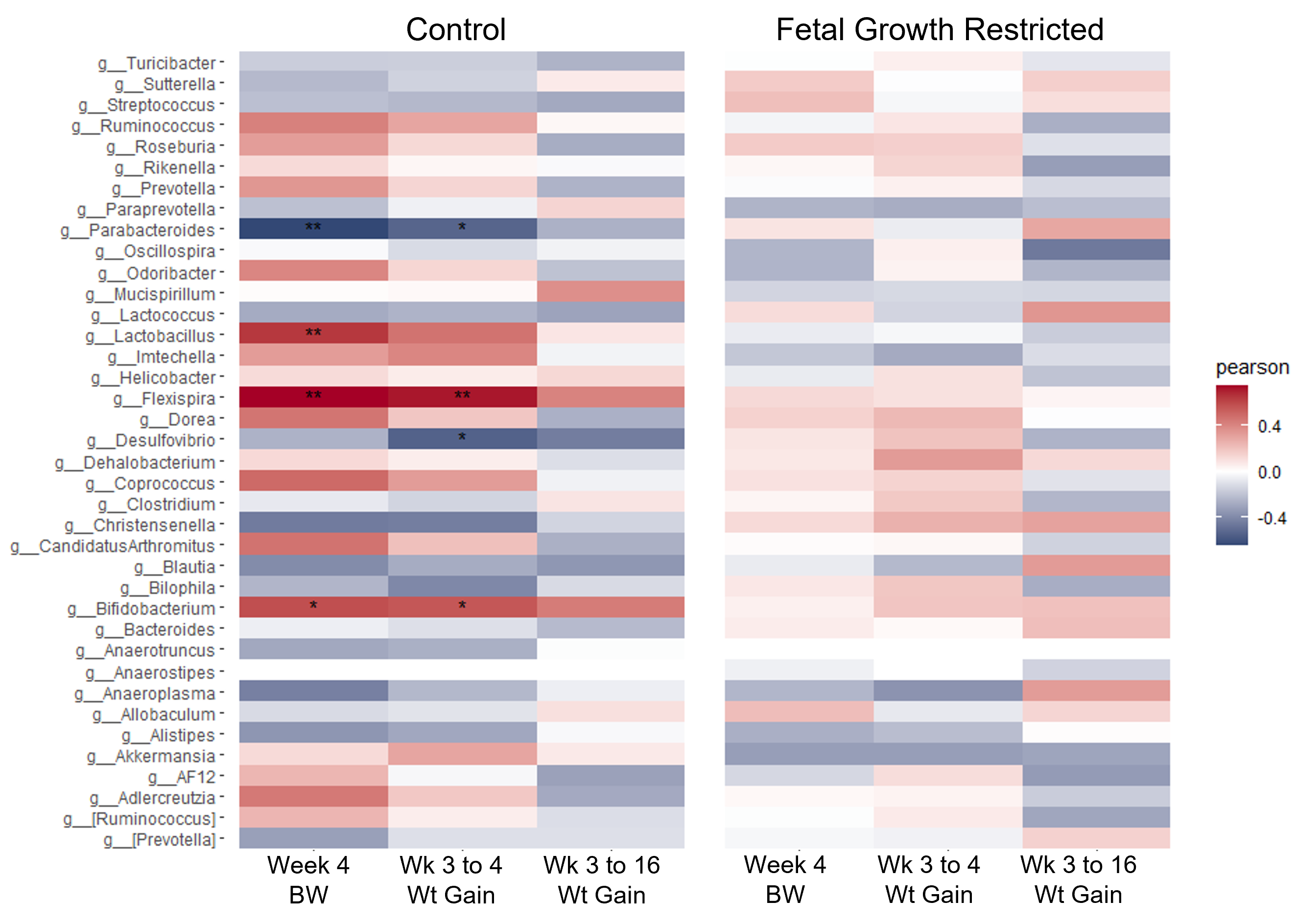

Supplement: Supplementary Figure S3 — Association of offspring genus-level microbial abundance at 4 weeks with weight outcomes without false discovery rate adjustment. Red-blue spectrum signifies positive to negative correlations, respectively. P values determined by Pearson correlations. *p < 0.05, **p < 0.01, ***p < 0.001, BW, body weight; wk, week; wt, weight. [file Image3.tif]

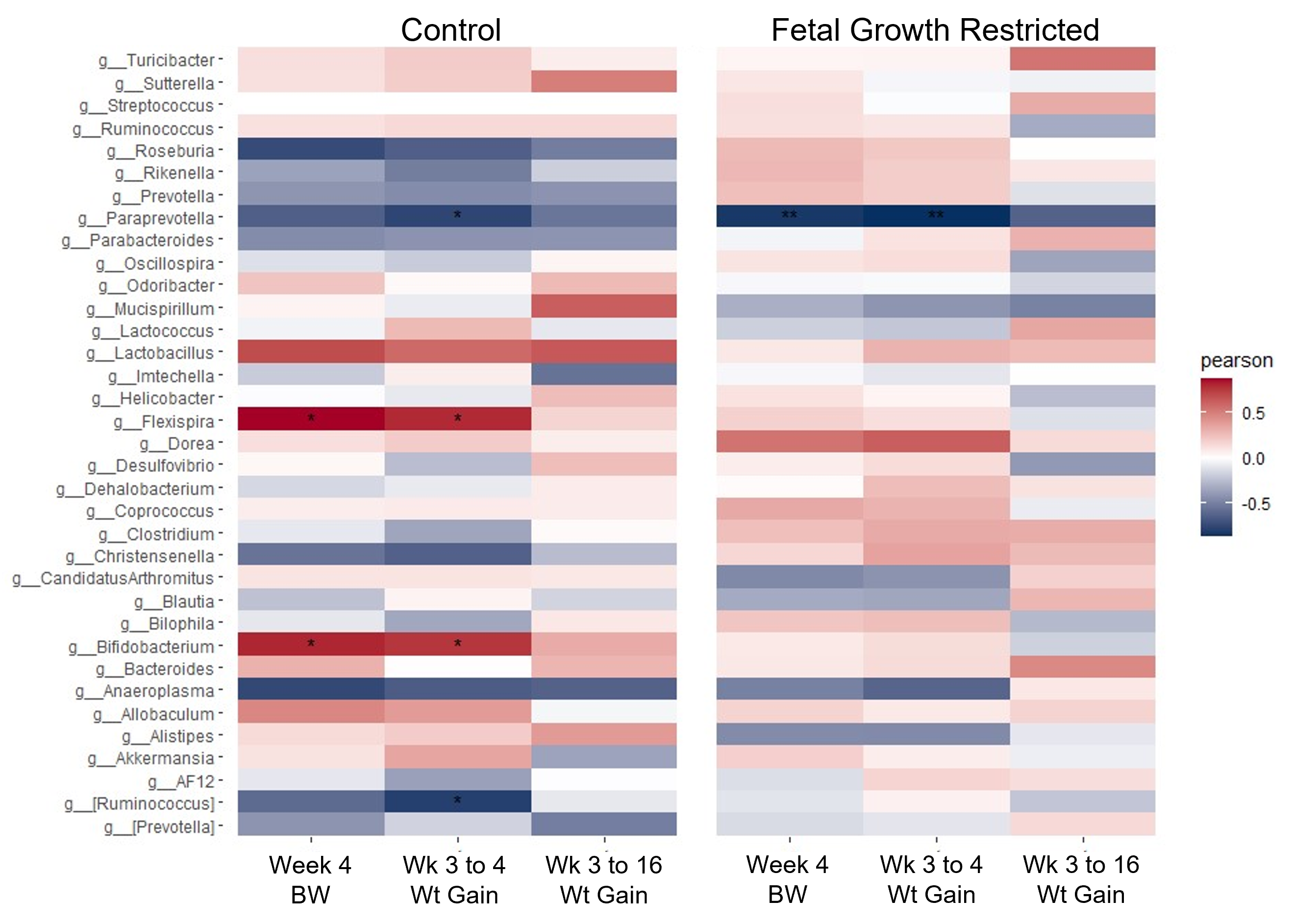

Supplement: Supplementary Figure S4 — Association of male offspring genus-level microbial abundance at 4 weeks with weight outcomes without false discovery rate adjustment. Red-blue spectrum signifies positive to negative correlations, respectively. P values determined by Pearson correlations. *p < 0.05, **p < 0.01, ***p < 0.001, BW, body weight. [file Image4.tif]

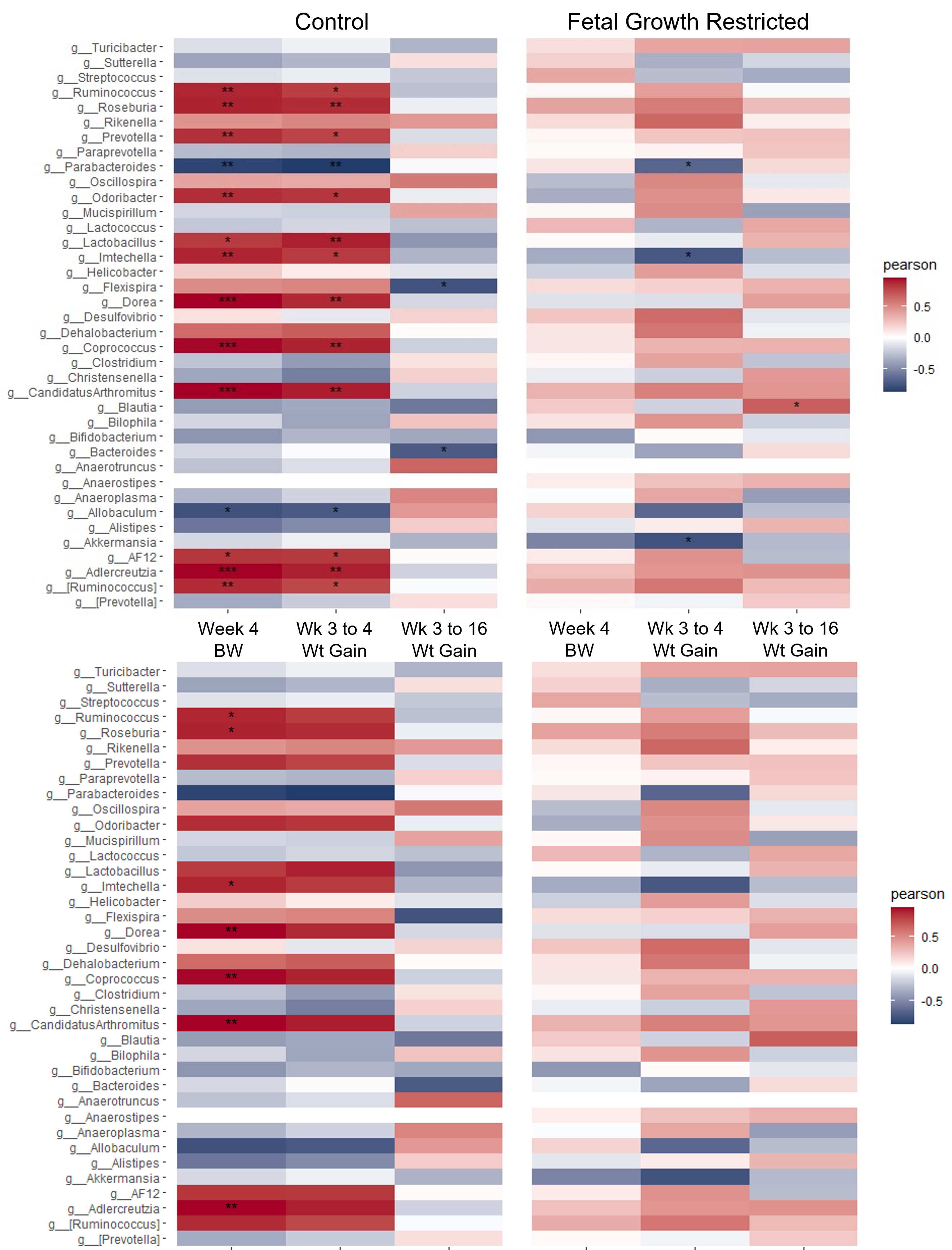

Supplement: Supplementary Figure S5 — Association of female offspring genus-level microbial abundance at 4 weeks with weight outcomes. Results are shown without (top) and with (bottom) False Discovery Rate Adjustment. Red-blue spectrum signifies positive to negative correlations, respectively. P values determined by Pearson correlations. *p < 0.05, **p < 0.01, ***p < 0.001, BW, body weight; wk, week; wt, weight. [file Image5.tif]
