# Supplementary material for: Calorie restriction during gestation impacts maternal and offspring fecal microbiome in mice
Source: Front Endocrinol (Lausanne). 2024 Oct 4;15:1423464. doi: 10.3389/fendo.2024.1423464 (PMC11487197; doi:10.3389/fendo.2024.1423464)
Supplement: Supplementary file 6 [file Table1.docx]

| **Male FGR Compared to Control Offspring at 3 weeks of age (n = 15 total)** | | | | | | | |  |  |
| --- | --- | --- | --- | --- | --- | --- | --- | --- | --- |
| **Phylum** | **Class** | **Order** | **Family** | **Genus** | **Coefficient** | **Std Dev** | **P value** | **Q value** | **# not zero** |
| **Decreased Abundance** | |  |  |  |  |  |  |  |  |
| Proteobacteria | Betaproteobacteria | Burkholderiales | Alcaligenaceae | Sutterella | -6.062 | 1.093 | **8.63E-04** | **0.026** | 11 |
|  |  |  |  |  |  |  |  |  |  |
| **Increased Abundance** | |  |  |  |  |  |  |  |  |
| Firmicutes | Clostridia | Clostridiales | Lachnospiraceae | Anaerostipes | 2.240 | 0.385 | **6.53E-04** | **0.025** | 3 |
| Bacteroidetes | Bacteroidia | Bacteroidales | Paraprevotellaceae | Paraprevotella | 7.693 | 2.607 | **0.021** | 0.365 | 10 |
|  |  |  |  |  |  |  |  |  |  |
| **Female FGR Compared to Control Offspring at 3 weeks of age (n = 13 total)** | | | | |  |  |  |  |  |
| **Phylum** | **Class** | **Order** | **Family** | **Genus** | **Coefficient** | **Std Dev** | **P value** | **Q value** | **# not zero** |
| **Decreased Abundance** | |  |  |  |  |  |  |  |  |
|  |  |  |  |  |  |  |  |  |  |
| **Increased Abundance** | |  |  |  |  |  |  |  |  |
| Firmicutes | Clostridia | Clostridiales | Ruminococcaceae | Oscillospira | 2.174 | 0.820 | **0.033** | 0.694 | 12 |
| Bacteroidetes | Bacteroidia | Bacteroidales | Rikenellaceae | Alistipes | 2.156 | 0.807 | **0.032** | 0.694 | 11 |

**Table S1: Sex-Stratified Analysis of Fecal Microbiome from Fetal Growth Restricted Offspring Compared to Control Offspring at 3 weeks of Age**

**Table S2: Sex-Stratified Analysis of Fecal Microbiome from Fetal Growth Restricted Offspring Compared to Control Offspring at 4 weeks of Age**

| **Male FGR Compared to Control Offspring at 4 weeks of age (n = 16 total)** | | | |  |  |  |  |  |  |
| --- | --- | --- | --- | --- | --- | --- | --- | --- | --- |
| **Phylum** | **Class** | **Order** | **Family** | **Genus** | **Coefficient** | **Std Dev** | **P value** | **Q value** | **# not zero** |
| **Decreased Abundance** | |  |  |  |  |  |  |  |  |
| Firmicutes | Erysipelotrichi | Erysipelotrichales | Erysipelotrichaceae | Allobaculum | -10.824 | 0.818 | **1.01E-06** | **3.54E-05** | 15 |
| Bacteroidetes | Bacteroidia | Bacteroidales | Porphyromonadaceae | Parabacteroides | -5.575 | 0.695 | **4.28E-05** | **0.001** | 16 |
| Bacteroidetes | Bacteroidia | Bacteroidales | Paraprevotellaceae | Paraprevotella | -6.167 | 0.914 | **0.000** | **0.002** | 13 |
| Firmicutes | Bacilli | Lactobacillales | Lactobacillaceae | Lactobacillus | -4.951 | 1.093 | **0.002** | **0.016** | 16 |
| Bacteroidetes | Bacteroidia | Bacteroidales | Paraprevotellaceae | Prevotella | -3.150 | 0.861 | **0.006** | **0.048** | 16 |
| Actinobacteria | Actinobacteria | Bifidobacteriales | Bifidobacteriaceae | Bifidobacterium | -5.026 | 1.769 | **0.022** | **0.121** | 15 |
| Bacteroidetes | Bacteroidia | Bacteroidales | Rikenellaceae | AF12 | -4.259 | 1.783 | **0.044** | **0.192** | 15 |
| Bacteroidetes | Bacteroidia | Bacteroidales | Rikenellaceae | Alistipes | -2.858 | 1.181 | **0.042** | **0.192** | 14 |
| Bacteroidetes | Bacteroidia | Bacteroidales | Bacteroidaceae | Bacteroides | -2.253 | 1.170 | 0.090 | **0.287** | 16 |
|  |  |  |  |  |  |  |  |  |  |
| **Increased Abundance** | |  |  |  |  |  |  |  |  |
| Firmicutes | Clostridia | Clostridiales | Lachnospiraceae | Roseburia | 4.429 | 0.474 | **1.41E-05** | **2.77E-04** | 4 |
| Firmicutes | Clostridia | Clostridiales | Lachnospiraceae | Dorea | 4.084 | 1.763 | **4.92E-02** | **0.212** | 6 |
| Firmicutes | Bacilli | Turicibacterales | Turicibacteraceae | Turicibacter | 3.090 | 1.364 | 0.053 | **0.221** | 4 |
|  |  |  |  |  |  |  |  |  |  |
| **Female FGR Compared to Control Offspring at 4 weeks of age (n = 17 total)** | | | |  |  |  |  |  |  |
| **Phylum** | **Class** | **Order** | **Family** | **Genus** | **Coefficient** | **Std Dev** | **P value** | **Q value** | **# not zero** |
| **Decreased Abundance** | |  |  |  |  |  |  |  |  |
| Proteobacteria | Deltaproteobacteria | Desulfovibrionales | Desulfovibrionaceae | Desulfovibrio | -5.798 | 0.711 | **3.79E-05** | **0.001** | 16 |
| Tenericutes | Mollicutes | Anaeroplasmatales | Anaeroplasmataceae | Anaeroplasma | -3.887 | 0.660 | **3.68E-04** | **0.007** | 8 |
| Verrucomicrobia | Verrucomicrobiae | Verrucomicrobiales | Verrucomicrobiaceae | Akkermansia | -3.838 | 0.762 | **0.001** | **0.015** | 4 |
| Bacteroidetes | Bacteroidia | Bacteroidales | Rikenellaceae | Rikenella | -4.041 | 0.928 | **0.002** | **0.026** | 13 |
| Proteobacteria | Deltaproteobacteria | Desulfovibrionales | Desulfovibrionaceae | Bilophila | -3.795 | 1.000 | **0.005** | **0.039** | 15 |
| Bacteroidetes | Bacteroidia | Bacteroidales | Rikenellaceae | AF12 | -1.566 | 0.662 | **0.046** | **0.175** | 17 |
| Firmicutes | Clostridia | Clostridiales | Dehalobacteriaceae | Dehalobacterium | -2.592 | 1.146 | 0.054 | **0.191** | 14 |
| Firmicutes | Clostridia | Clostridiales | Ruminococcaceae | Oscillospira | -3.238 | 1.509 | 0.064 | **0.213** | 17 |
|  |  |  |  |  |  |  |  |  |  |
| **Increased Abundance** | |  |  |  |  |  |  |  |  |
| Firmicutes | Clostridia | Clostridiales | Lachnospiraceae | Dorea | 3.360 | 0.930 | **0.007** | **0.045** | 10 |
| Firmicutes | Bacilli | Lactobacillales | Lactobacillaceae | Lactobacillus | 1.946 | 0.670 | **0.020** | **0.096** | 17 |
